# Immunological Parameters Associated With Vitiligo Treatments: A Literature Review Based on Clinical Studies

**DOI:** 10.1155/2015/196537

**Published:** 2015-09-17

**Authors:** Ana Cláudia Guimarães Abreu, Gabriela Guy Duarte, Juliana Yasmin Pains Miranda, Daniel Gontijo Ramos, Camila Gontijo Ramos, Mariana Gontijo Ramos

**Affiliations:** ^1^Faculdade de Ciências Humanas, Sociais e da Saúde, Universidade Fumec, 30310-190 Belo Horizonte, MG, Brazil; ^2^Departamento de Dermatologia, Santa Casa de Belo Horizonte, 30150-221 Belo Horizonte, MG, Brazil; ^3^Clínica de Dermatologia, Edificio Life Center, 30110-921 Belo Horizonte, MG, Brazil

## Abstract

Vitiligo, a depigmentary disorder, caused by the loss of melanocytes, affects approximately 1% of the world population, irrespective of skin type, with a serious psychological impact on the patient quality of life. So far, the origin of vitiligo has not been traced and the pathogenesis is complex, involving the interplay of a multitude of variables. Although there is no treatment that ensures the complete cure of the disorder, there are some pharmacological, phototherapy, and surgical therapies available. A series of variables can affect treatment outcome, such as individual characteristics, emotional issues, type of vitiligo, stability of the lesions, and immunological status. The present literature review identified the main immunological parameters associated with treatments for vitiligo. Cytotoxic CD8+ T lymphocytes are the main cell type involved in treatment success, as fewer cells in skin lesions are associated with better results. Other parameters such as cytokines and regulatory T cells may also be involved. Further clinical scientific studies are needed to elucidate the complex mechanisms underlying vitiligo and its treatments, in order to expand the range of therapeutic approaches for each individual case.

## 1. Introduction

Vitiligo is an acquired, usually asymptomatic pigmentary disorder that results in the loss of functional melanocytes and is often associated with other autoimmune diseases. At the onset of the disease white patches of different sizes appear on different parts of the body [1, 2]. Vitiligo affects approximately 1% of the world population of all skin types, usually before the age of 20 [3]. Its psychological impact on the quality of life can be disastrous, as dissatisfaction with body image can smother self-esteem and develop a depressive state, especially among dark or tan-skinned patients [4]. The course of the disease is unpredictable, with peaks of anxiety, which makes the patient feel an urge to try different types of treatments. The fact that it can be rather hard to hide the white patches from the eyes of other people makes it difficult for the patient to cope with the disease on a daily basis [5, 6]. Vitiligo can be clinically classified as follows: Nonsegmental or generalized vitiligo is a group that includes acrofacial, vulgaris, universalis, and mixed forms. Localized vitiligo can affect one, two, or multiple segments and includes focal, segmental, and mucosal forms. There are also mixed and undetermined forms of vitiligo [7, 8].

## 2. Pathogenesis of Vitiligo

Vitiligo is an intriguing disorder whose cause has been an extensive topic of debate. The exact origin of vitiligo is still unclear, and the pathogenesis is complex and involves the interplay of a series of variables [9–11]. There is a multifactorial genetic component predisposing certain individuals to vitiligo and family history is a variable found in approximately one-third of the people with the disease [3, 7]. There is also strong genetic evidence of a link between vitiligo and other autoimmune diseases [12].

According to the neural theory, segmental vitiligo follows the same path as dermatome, and dysfunction of the sympathetic nervous system can curb melanin production and lead to depigmentation [8].

The intrinsic theory suggests that defects in vitiligo melanocytes lead to their death. These include morphologic defects, decreased adhesive properties, and deficient melanocyte growth factors [13, 14]. Increased oxidative stress has also proved to be an important cause for melanocytes destruction [15, 16].

The theory of autoimmune mediated destruction of melanocytes is well accepted and seems to have currently become the leading hypothesis in vitiligo pathogenesis. The immune reaction can be mediated by cellular immunity, humoral antibody-mediated immunity, and the action of cytokines [8].

The action of antibodies against different melanocyte-associated antigens was confirmed in vitiligo. The main antigen recognized by these antibodies is tyrosinase, but antibodies against tyrosine hydroxylase, pigment cell surface antigens, and antithyroid antigens have also been found [17, 18].

Cell-mediated immunity in vitiligo is demonstrated by the presence of inflammatory infiltrates in perilesional vitiligo skin. Decreased CD4+ to CD8+ lymphocytes ratio in vitiligo-stricken skin compared to healthy skin and CD8 T cells directed against melanocytic antigens have been found both in perilesional skin and in the blood of vitiligo patients [19–21]. This shows that the elimination of melanocytes by cytotoxic T cells is a mechanism leading to depigmentation in vitiligo.

Cytokines also seem to play an important role in vitiligo pathogenesis. There is an increase in the expression of tumor necrosis alpha (TNF-*α*) and interferon-gama (IFN-*γ*), suggesting that vitiligo is mediated by a T helper cell-1 (Th1) response [22].

The role of regulatory T cells in vitiligo pathogenesis has been a recent topic of research, and there is evidence that Treg is jeopardized in vitiligo patients. However, there is some contradicting data and this subject demands further investigation [8].

Given the importance of the immune system in depigmentation lesions in vitiligo, a series of treatments meant for altering or decreasing immunological reactions has been developed.

## 3. Vitiligo Treatment

There are no treatments ensuring the complete cure of vitiligo. The main goal of the current treatments is either to control the autoimmune destruction of melanocytes or stimulate their growth on affected areas. Treatment can encompass pharmacological, physical, and surgical approaches or still a combination of different procedures [7].

Pharmacological treatment consists of a topical and systemic corticosteroid therapy, topical calcineurin inhibitors such as tacrolimus and pimecrolimus, pseudocatalase, melagenina (human placental extract), and vitamin-D derivatives [3, 7].

Light therapy includes sunlight, PUVA, narrowband UVB (NB-UVB), and excimer laser. These treatments are frequently combined with topical treatment and transplantation processes [23].

**Figure 1:**
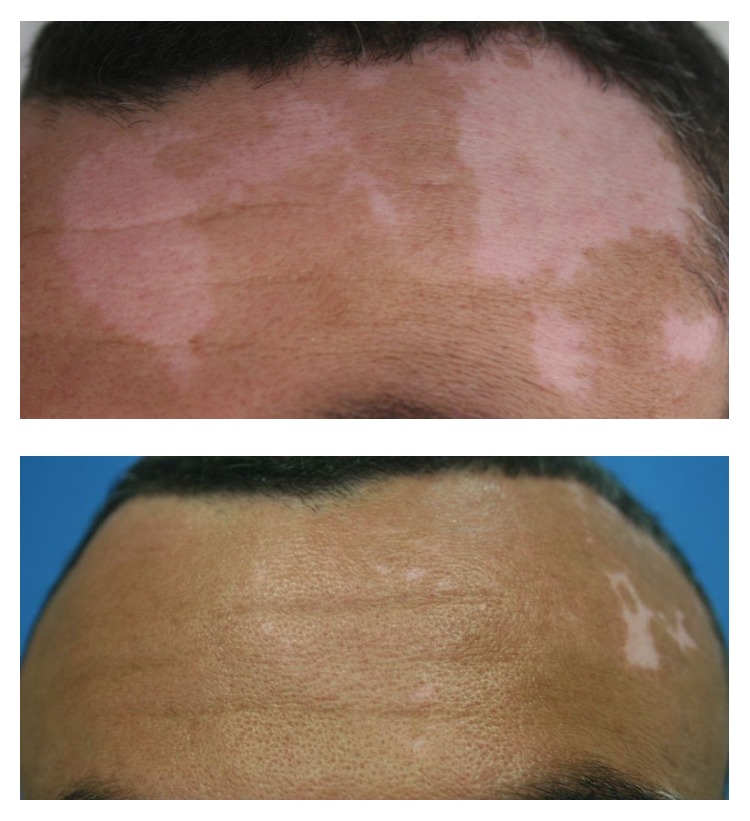
Repigmentation observed after melanocyte transplantation for the treatment of stable vitiligo.

Surgical treatment can be an option and is usually recommended for patients with stable vitiligo (with no change in lesions numbers or morphology), for which other treatments had no significant results. There are various techniques, but the most usual technique consists of transplanting a pigmented skin graft from a donor onto the patient's affected areas. Surgical treatments include suction blister grafts, punch grafts, minigrafts, and split thickness skin grafts [24, 25]. Melanocytes and keratinocytes transplantation is also a surgical intervention by which cell suspension is transferred from a donor's pigmented skin onto a previously prepared recipient, usually by means of dermabrasion. Cultured and noncultured cells can be used [26–28]. Noncultured melanocytes-keratinocytes cellular grafting yields significant pigmentation results in patients with stable forms of vitiligo, as seen from data previously collected by our group (Figure 1) [29].

Although there are several options for the treatment of vitiligo, the results are different for each patient and hardly ever satisfying, as these people can be affected by a series of unknown factors. Knowledge of the immune parameters associated with vitiligo pathogenesis is therefore vital in predicting and suggesting the right type of treatment for each patient.

## 4. Immunological Parameters Associated with Vitiligo Treatments

Different treatments for vitiligo can lead to good repigmentation and great satisfaction for some patients. However, they may not work so well for others. Treatment outcome will depend on some rather complex mechanisms, such as individual characteristics, emotional issues, type of vitiligo, stability of the lesions, and immunological status.

Although there are many studies showing the importance of the immune reaction to the pathogenesis of vitiligo, few of them were conducted in vivo in human patients submitted to treatments.

The purpose of the present literature review is to investigate and analyze clinical studies about the association of immunological parameters with treatments for vitiligo. Data collected from Pubmed database suggested that there is a growing interest in this subject, as most of the studies have been published quite recently, after 2010. It was also observed that research groups are mainly from Asian countries, such as India and China. The main findings of the studies are summarized in Table 1.

Most of the patients enrolled in the studies suffered from generalized vitiligo and were submitted to different types of treatments, such as NB-UVB, PUVA alone or combined with* Polypodium leucotomos,* alternative treatment with Chinese herbs or minigrafts, surgical treatment, or autologous melanocytes transplantation.

The importance of the patient's immunological status for the outcome of vitiligo treatments was observed in all studies.

Cellular immunity seems to play a vital role in the outcome of vitiligo treatments, as CD8+ T cells are involved in the autoimmune destruction of melanocytes resulting in skin depigmentation. In 2012, Rao et al. reported that patients with active vitiligo, when compared to patients with stable vitiligo, presented increased numbers of CD8+ and CD45RO+ cells (effector lymphocytes) on perilesional skin and had the worst response to the treatment with melanocyte transplantation [30]. A similar result showing increased cytotoxic T lymphocytes in vitiligo skin lesions and poor response to minigraft transplantation was demonstrated by Abdallah et al. [31].

Zhou et al. treated patients with melanocyte transplantation and observed an increased number of CD8+ lymphocytes in the perilesional skin of patients with poor repigmentation results. They also demonstrated that dermal mesenchymal stem cells (DMSC) had an inhibitory effect against CD8+ T cells skin-homing, inhibited cell proliferation, induced apoptosis, and regulated the production of cytokines and chemokines by CD8+ lymphocytes [32]. A decrease in peripheral blood CD8+ lymphocytes can be associated with the increased homing of these cells to vitiligo-affected skin sites; once in the active disease the lymphocytes migrate to the skin, due to cutaneous lymphocyte-associated antigen (CLA) molecule expression [33, 34].

Alternative treatments, which can be administered alone or in combination with other procedures, are sometimes an option for patients who are unresponsive to regular treatments. Oral administration of Chinese herbs (Zengse pill) combined with oral cobalamin and topic psoralea tincture induced significant repigmentation and increased blood CD4+/CD8+ ratio, regulating the immunity of the organism [35].

Treatment with PUVA alone has proved inefficient and incapable of altering immunologic parameters in patients [34]. However, a combination of PUVA and* Polypodium leucotomos* succeeded in inducing repigmentation and curbed the proliferation of lymphocytes in patients with generalized vitiligo [33].

Regulatory T cells (Treg) are lymphocytes specialized in self-tolerance and in preserving immune system homeostasis. These cells are responsible for the production of modulatory cytokines. There have been advances in the research on Treg cells in the pathogenesis of vitiligo and on successful treatments. Regulatory cytokines produced by Treg cells, such as interleukin-10 and tumor growth factor beta (TGF-*β*), are suggested to be related to the stability of the disease. In 2013, Tembhre et al. found increased cytokine levels in patients with stable vitiligo, and treatment with NB-UVB was capable of elevating TGF-*β* levels, suggesting that Treg cytokines played an important role in repigmentation [36]. However, no evidence of a correlation between Treg and duration or activity of vitiligo was found by Moftah et al. in 2014, indicating that more studies are needed to further elucidate these mechanisms [37].

Other cytokines are probably involved in vitiligo pathogenesis and treatment. Higher levels of the proinflammatory cytokines Interleukin-1*α*, Interleukin-1*β*, and interleukin-12, measured in epidermis fluid, were present in patients whose response to melanocyte transplantation was unsatisfactory [32]. Patients with active vitiligo also showed an increase in blood proinflammatory cytokines [36]. These data suggest that an increased inflammatory response mediated by Th1 cytokines is associated with disease activity and consequently poor skin repigmentation.

It is common knowledge that stable vitiligo can help predict response to different treatments, mainly surgical procedures for the replacement of melanocytes in depigmented skin sites. The course of the disease is difficult to predict, as it can either stay stable for years and then become active again or regress spontaneously. Literature review indicates that more stable lesions present lower numbers of cytotoxic T cells and inflammatory cytokines, leading to a better prognosis [30, 36].

Regarding the type of vitiligo, it is possible to conclude that patients with the generalized form of the disease will have the worst response to different treatments, compared to those with segmental vitiligo. An explanation for this observation is the fact that generalized vitiligo patients have more lesions in many body areas and thus need more aggressive and long lasting treatments.

## 5. Conclusions

Literature review shows that immunological parameters, mainly cytotoxic T lymphocytes, cytokines, and regulatory T cells, can influence the outcome of different treatments for vitiligo. Lower levels of CD8+ cells in skin lesions are usually associated with better repigmentation results. However, further scientific studies and efforts are needed, especially through clinical studies, to elucidate the complex mechanisms underlying vitiligo and its treatments, leading to better therapeutic choices for each individual case.

## Figures and Tables

**Table 1:** Summary of immunological parameters associated with vitiligo treatments reported in the literature.

Author	Type of vitiligo	Treatment	Main findings in immunological parameters	Reference
Rao et al.	Generalized	Surgical by suction blister epidermal grafting	Patients with active vitiligo and poor treatment response had increased levels of CD8+ and CD45RO+ cells in skin lesions	[30]

Abdallah et al.	Generalized	Surgical by autologous minigrafting	Patients unresponsive to treatment had increased cytotoxic T lymphocyte in skin lesions	[31]

Zhou et al.	Localized and generalized	Surgical by autologous melanocyte transplantation	Patients with poor repigmentation response had higher levels of CD8+ T cells in perilesional skin and increased levels of proinflammatory cytokines in epidermis fluid	[32]

Reyes et al.	Generalized	PUVA and *Polypodium leucotomos*	Abnormal activation and decrease in CD8+CD45RO+ blood lymphocytes	[33]

Antelo et al.	Generalized	PUVA	Reduction of CD8+CLA+ lymphocytes in peripheral blood	[34]

Shi et al.	Vitiligo with qi-stagnancy and blood-stasis	Chinese herbs Zengse pill combined with cobalamin and psoralea tincture	Increased CD4/CD8 ration and reduced CD8+ in peripheral blood	[35]

Tembhre et al.	Generalized	NB-UVB	Increased serum IL-10, IL-13, and IL-17 and decreased TGF-*β* in active vitiligo patients. NB-UVB may be able to modulate T helper and Treg cytokines.	[36]

Moftah et al.	Generalized	NB-UVB	NB-UVB treatment decreased peripheral blood Treg cells.	[37]
